# Kinetochore-Independent Chromosome Poleward Movement during Anaphase of Meiosis II in Mouse Eggs

**DOI:** 10.1371/journal.pone.0005249

**Published:** 2009-04-13

**Authors:** Manqi Deng, Juntao Gao, Praveen Suraneni, Rong Li

**Affiliations:** Stowers Institute for Medical Research, Kansas City, Missouri, United States of America; University of Edinburgh, United Kingdom

## Abstract

Kinetochores are considered to be the key structures that physically connect spindle microtubules to the chromosomes and play an important role in chromosome segregation during mitosis. Due to different mechanisms of spindle assembly between centrosome-containing mitotic cells and acentrosomal meiotic oocytes, it is unclear how a meiotic spindle generates the poleward forces to drive two rounds of meiotic chromosome segregation to achieve genome haploidization. We took advantage of the fact that DNA beads are able to induce bipolar spindle formation without kinetochores and studied the behavior of DNA beads in the induced spindle in mouse eggs during meiosis II. Interestingly, DNA beads underwent poleward movements that were similar in timing and speed to the meiotic chromosomes, although all the beads moved together to the same spindle pole. Disruption of dynein function abolished the poleward movements of DNA beads but not of the meiotic chromosomes, suggesting the existence of different dynein-dependent and dynein-independent force generation mechanisms for the chromosome poleward movement, and the latter may be dependent on the presence of kinetochores. Consistent with the observed DNA bead poleward movement, sperm haploid chromatin (which also induced bipolar spindle formation after injection to a metaphase egg without forming detectable kinetochore structures) also underwent similar poleward movement at anaphase as DNA beads. The results suggest that in the chromatin-induced meiotic spindles, kinetochore attachments to spindle microtubules are not absolutely required for chromatin poleward movements at anaphase.

## Introduction

Accurate chromosome segregation during eukaryotic cell division is achieved by a microtubule-based bipolar spindle which generates forces to move the replicated chromosomes toward opposite poles. It has been a long standing question how spindle microtubules (as well as the associated motor molecules) generate the poleward forces to segregate chromosomes during cell division. Although the detailed molecular mechanisms are still not completely understood, it is generally accepted that the kinetochore, a proteinaceous structure assembled at the chromosomal centromere region, plays important roles in chromosome segregation [1], [2], [3], [4], [5], [6], [7]. The kinetochore mediates the physical interactions between a replicated chromosome and microtubules to establish a biorientation configuration during metaphase and ensure accurate segregation during anaphase [4], [5], [8], [9], [10]. The kinetochore-connected microtubules, or K-fibers, are thought to apply the poleward forces to the chromosomes and pull chromosomes toward the opposing poles during anaphase [11], [12], [13].

In addition to the forces exerted on the kinetochores, it is known that the chromosome arms are associated with motor molecules, which can also generate forces to maintain chromosome mobility [14], [15]. Most of them however, are plus end-directed motors responsible for generating the “polar ejection” forces to help position chromosomes at the spindle equator during chromosome congression [14], [16], [17]. The minus end-directed motor, dynein, localizes mainly to the kinetochores and the spindle poles [18], [19]. It was previously observed in plant cells and insect spermatocytes that chromosome fragments (made by cutting chromosomes) containing no kinetochores still show poleward movement during both metaphase and anaphase [20], [21], suggesting that there are kinetochore-independent poleward forces exerted on the chromosome arms.

Meiotic spindles in mammalian oocytes show some fundamental differences from their mitotic counterparts and little is known about the force generation mechanism in the meiotic spindle to move chromosomes from spindle equator to poles. Oocytes contain no centrosomes [22], [23] and the meiotic spindle is assembled around the chromatin via a Ran^GTP^-dependent pathway [24], [25], [26]. It is interesting to note that the K-fibers are not involved in the formation of the first meiotic spindle or meiotic chromosome congression to the metaphase plate [27]. More impressively, a morphologically indistinguishable bipolar spindle can be induced by DNA coated beads in *Xenopus* egg extracts [28] and mouse eggs [29], suggesting a distinct centrosome-independent pathway for meiotic spindle formation. Microtubules can even self-organize into bipolar spindles in the absence of chromatin [29], [30] through motor-driven processes [28], [31]. Apart from being useful for studying the mechanism of spindle formation, DNA bead-spindles may provide a clean kinetochore-null system for observation and dissection of the poleward forces exerted on the chromosomes. Another advantage of studying anaphase chromosome movement in mouse oocytes is that the anaphase of meiosis II can be experimentally induced and manipulated without imposing cell cycle arrest. In this study, we injected DNA beads into mouse eggs to induce spindle formation without kinetochores and studied the behavior of DNA beads after inducing anaphase of meiosis II. To our surprise, DNA beads underwent progressive poleward movements as was observed for the meiotic chromosomes. The kinetochore-independent poleward movement of DNA beads required dynein and dynamic microtubules, while the poleward movement of meiotic chromosomes which contain kinetochores was independent of dynein. Our findings reveal a kinetochore-independent mechanism to drive chromosome poleward movement during meiosis.

## Results

### DNA beads undergo poleward movements during anaphase of meiosis II

**Figure 1:**
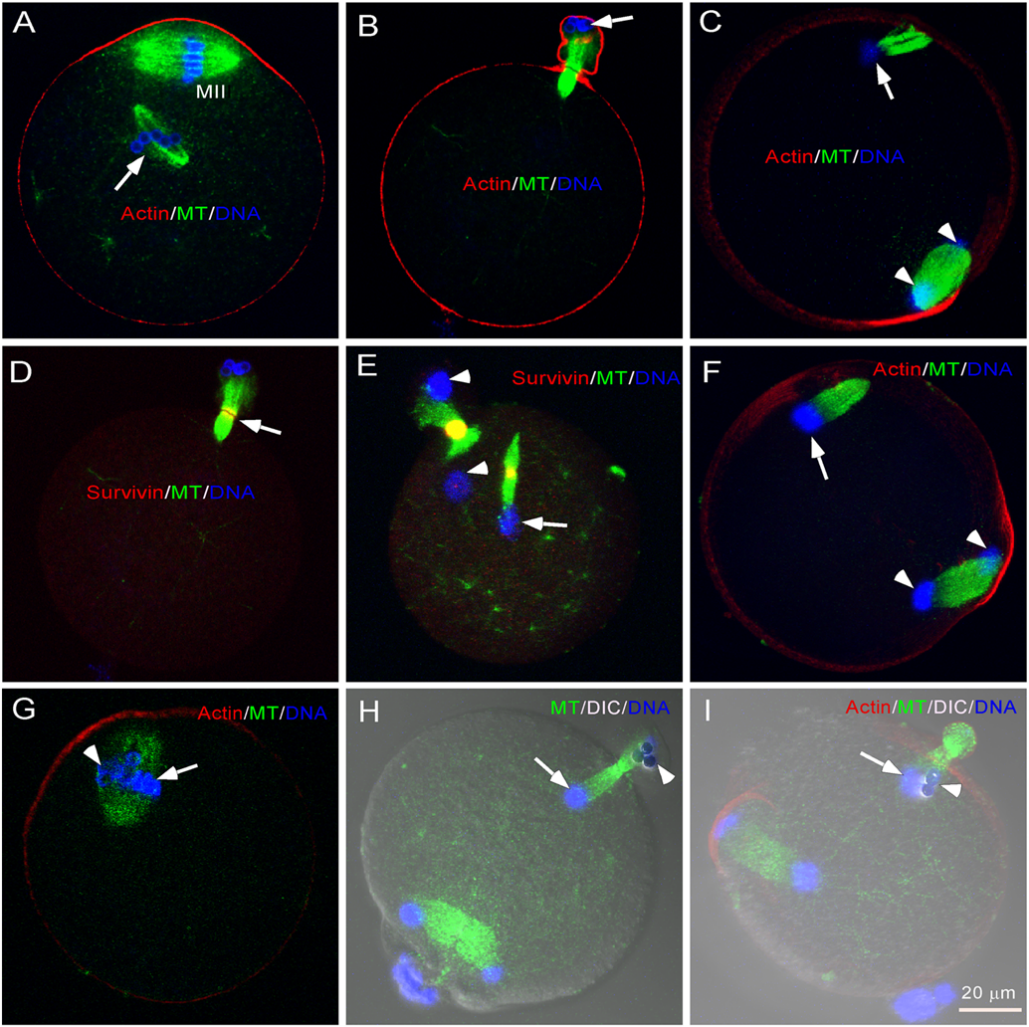
Behavior of DNA beads and sperm chromatin in their assembled K-fiber-less spindles during anaphase of meiosis II. (A) A DNA bead-assembled bipolar spindle (arrow) in MII eggs (MII, maternal chromosome-induced spindle). (B) DNA bead-spindle underwent metaphase-anaphase transition and induced polar body extrusion after egg activation. Note that the DNA beads were moved to one spindle pole and discarded in the extruded polar body (arrow). (C) DNA bead and meiotic chromosome poleward movements preceded the polar body extrusion. The arrow indicates the DNA beads and arrowheads show the segregated meiotic chromosomes. Note that the polar body was not yet extruded in this egg. The arrowheads show the segregated meiotic chromosomes. (D) Survivin (in red) localization at the spindle midzone (arrow) of the DNA bead-spindle (the same egg shown in panel B). (E) DNA bead poleward movement on a spindle formed far away from the cortex. The arrow indicates the DNA beads and the arrowheads indicate the two sets of segregated meiotic chromosomes. (F) Poleward movement of injected sperm chromatin to one spindle pole (arrow). The arrowheads show the segregated meiotic chromosomes. (G) Co-injected DNA beads (arrowhead) and sperm chromatin (arrow) sharing the same metaphase spindle. (H) After anaphase onset, DNA beads (arrowhead) and sperm chromatin (arrow) segregated from each other and moved to opposite poles in 7/20 oocytes. The unmarked spindle is maternal. (I) Both DNA beads (arrowhead) and sperm chromatin (arrow) moved to the same spindle pole in 13/20 oocytes. The unmarked spindle is maternal.

To investigate the behavior of chromatin in the absence of kinetochores in an anaphase spindle, we injected beads coated with plasmid DNA into mouse eggs which are physiologically arrested at metaphase of the second meiotic division (MII), to induce bipolar spindle formation [28], [29]. The injected eggs, each containing a maternal spindle and a DNA bead-induced spindle (Figure 1A, arrow), were either activated parthenogenetically by using SrCl_2_ [32], [33] or by *in vitro* fertilization [34] to induce anaphase onset. After egg activation, the DNA bead-spindles underwent metaphase-anaphase transition, forming a distinct spindle midzone, a structure typical of an anaphase spindle (Figure 1D, arrow), which was capable of inducing cytokinesis for polar body extrusion (Figure 1B). Interestingly, all the DNA beads moved together to one spindle pole, either toward the polar body or remained inside the egg (Figure 1B–1C, arrows). The observed poleward movement of DNA beads was not due to spindle-cortex interaction or cortical attraction during polar body extrusion because: 1) the poleward movement preceded the polar body extrusion (Figure 1C, movie S1); and 2) DNA bead-spindles that formed far away from the cortex showed no difference from those close to the cortex in moving DNA beads poleward (Figure 1E, arrow).

**Figure 2:**
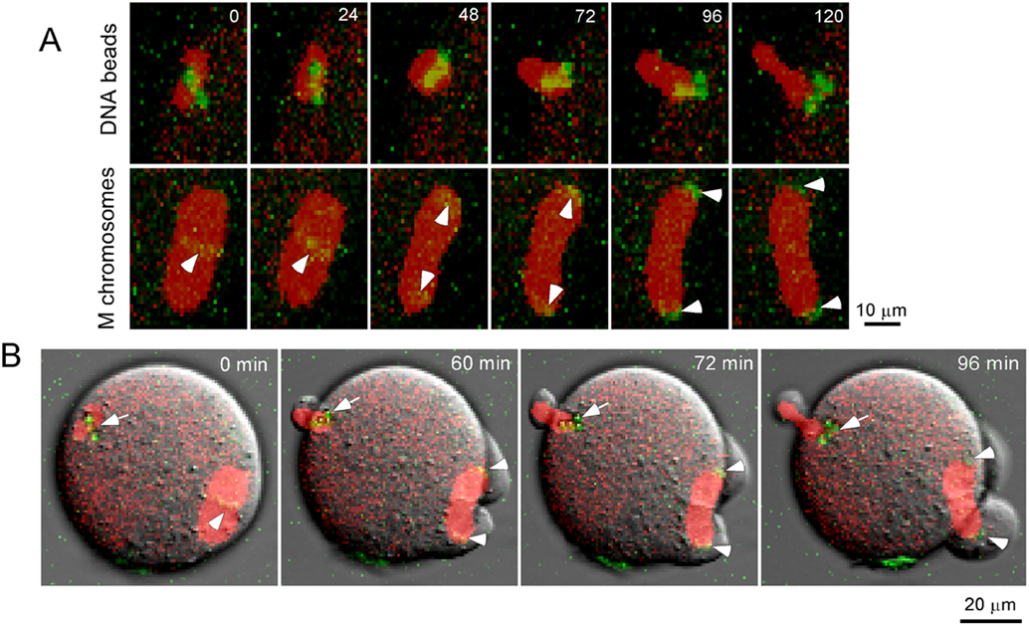
Time-lapse 3D confocal observation of chromosome poleward movements during anaphase of meiosis II. (A) Movement of DNA beads (top) and meiotic chromosomes (lower) at different time points shown in minute in a timelapse 3D movie. M chromosomes represent maternal chromosomes. DNA beads and meiotic chromosomes are shown in green and spindle in red. Note that the apparent round morphology of DNA-bead spindle shown at 48 min was due to a slight spindle rotation in the Z dimension. (B) Entire oocyte showing synchronous anaphase events (including anaphase onset, chromosome poleward movement, spindle rotation, and polar body extrusion) in both the DNA bead-spindle (arrows) and the meiotic spindle (arrowheads).

To compare the rates of poleward movement of the DNA beads with that of the maternal chromosomes, time-lapse 3D confocal movies were made to follow the anaphase events (Figure 2, Supplementary Figure S1). First, we observed that the poleward movements of DNA beads and maternal chromosomes occurred almost synchronously after egg activation, suggesting that the forces that drove DNA bead movement were under the same cell cycle control as those moving the maternal chromosomes. Second, transition from metaphase to anaphase was coupled with re-orientation of the spindle such that the spindle was positioned ∼90° to the polar cortex where the polar body is extruded. Using Imaris 4D tracking software, the rate of poleward movement was calculated to be 0.24±0.06 µm/min (n = 3) and 0.28±0.06 µm/min (n = 3). Thus, DNA beads underwent poleward movement at a speed similar to that of the meiotic chromosomes.

It should be pointed out that DNA beads that were incorporated into the metaphase spindle showed no obvious poleward movements and stayed in the equator of the spindle during metaphase (observation of over 50 DNA bead-spindles). The poleward movement of DNA beads was only observed after anaphase onset.

### Sperm chromatin moves towards spindle poles in the absence of K-fibers

**Figure 3:**
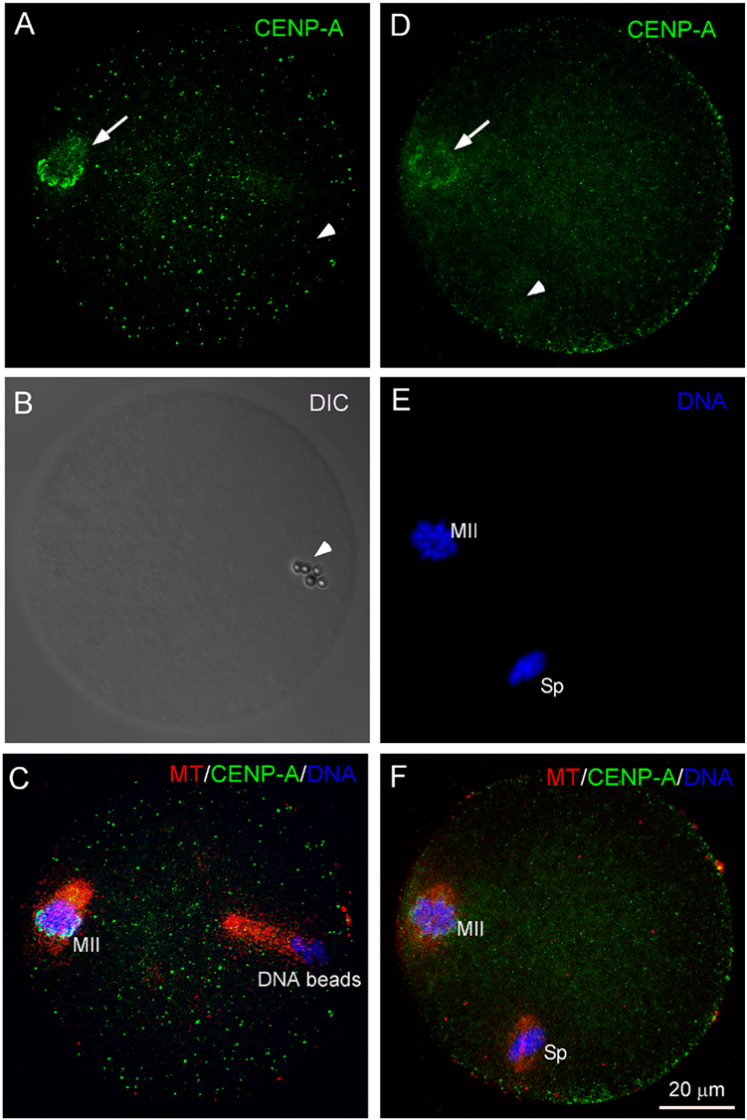
Lack of CENP-A in DNA bead- and sperm chromatin-assembled spindles in MII eggs. (A) CENP-A in a DNA bead-injected egg. The arrow indicates the location of MII chromosomes and the arrowhead indicates the position of DNA beads. (B) DIC image of (A) showing the position of the injected DNA beads (arrowhead). (C) The same egg of (A) showing spindle (red) and CENP-A (green) and DNA (blue). Note that the two spindle poles induced by the DNA beads are not in the same focal plane. (D–F) A sperm chromatin-injected egg showing CENP-A (green), DNA (blue) and spindle (red). The arrow indicates the location of MII chromosomes and the arrowhead indicates the location of the injected sperm chromatin. MII represents the meiotic chromosomes and Sp indicates sperm chromatin.

To test if the observed DNA bead poleward movement is a general phenomenon of kinetochore-free chromosomes during meiosis, we injected mouse sperm chromatin into eggs to induce spindle formation and followed their behavior during meiosis II. It was previously observed that *Xenopus* sperm chromatin is able to induce spindle formation in egg extracts without forming kinetochores or kinetochore microtubules [35]. This was confirmed by negative immunostaining of centromere protein A (CENP-A), a kinetochore marker, in both DNA bead- and sperm-assembled spindles (Figure 3A, 3D, arrowheads). Consistent with that observed in DNA bead-spindles, the condensed sperm chromatin moved poleward without disjunction during anaphase of meiosis II (Figure 1F, arrow). The monopolar movements of DNA beads and sperm chromatin might be due to physical stickiness of the beads or the highly condensed sperm chromatin. To test this possibility, DNA beads were co-injected with sperm chromatin into an egg to induce formation of a single spindle containing both DNA beads (Figure 1G, arrowhead) and sperm chromatin (Figure 1G, arrow). After inducing anaphase onset, the co-injected DNA beads and sperm chromatin either segregated from each other and moved toward opposite poles (Figure 1H, arrow and arrowhead, n = 7/20), or moved together to the same pole (Figure 1I, arrow and arrowhead, n = 13/20). This suggests that in the absence of kinetochore, the chromosome-induced spindle has the ability to move chromosomes toward opposite poles but loses the assurance of equal segregation.

### Disruption of dynein function abolishes the poleward movement of DNA beads but not of the meiotic chromosomes

**Figure 4:**
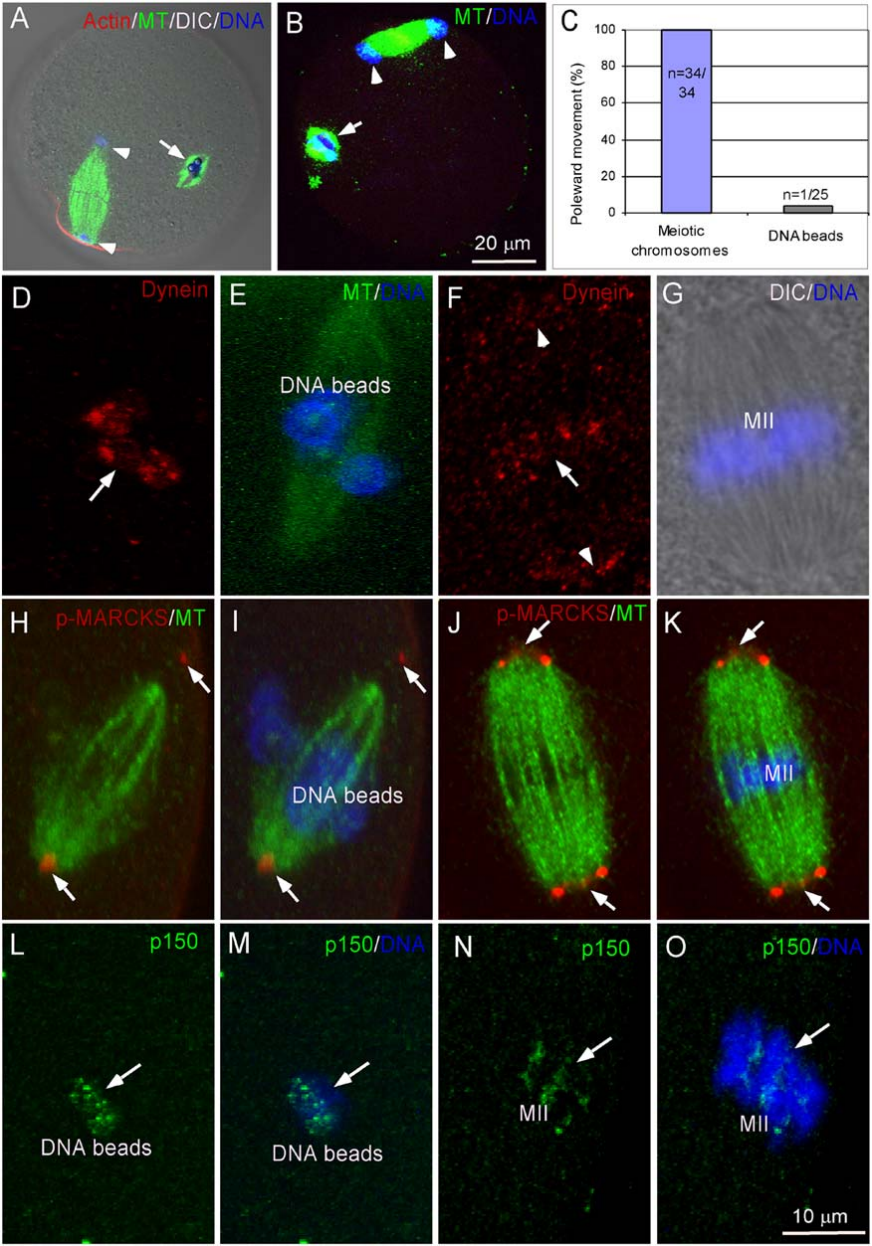
Role of cytoplasmic dynein in chromosome poleward movement. (A) Inhibition of poleward movement of DNA beads and maternal chromosomes by dynein antibody injection. (B) Injection of dynamitin p50. Note that DNA beads remained at the middle of the spindles (arrows) whereas the maternal chromosomes segregated and moved to spindle poles (arrowheads). (C) Quantification of the percentage of chromosome poleward movements on the meiotic and DNA bead spindles respectively in the dynein antibody-injected eggs. (D) Dynein localization on DNA beads (arrow). (E) Merged image of DNA beads (blue) and spindle (green). (F) Dynein localization on MII chromosomes (arrow) and spindle poles (arrowheads). (G) Merged image of DIC and DAPI staining showing MII chromosomes (blue) and spindle. (H–K) p-MARCKS staining of microtubule organization centers (MTOCs, red) in DNA bead spindle (H and I) and MII spindle (J and K). Note that there is only one MTOC staining in each of the DNA bead-spindle poles (H, arrows). (L–O) p150Glued localization on both DNA beads (H and I, arrows) and meiotic chromosomes (J and K, arrows).

To gain insights into the forces responsible for driving the kinetochore-independent poleward movements of DNA beads during anaphase, we tested the potential role of two microtubule motors using chemical or protein inhibitors. Whereas we were unable to obtain conclusive results with inhibition of Eg5 kinesin by monastrol due to severe defects in maintaining a bipolar spindle morphology in anaphase (data not shown), we found that dynein, a minus end-directed microtubule motor, was required for the anaphase poleward movement of DNA beads, as injection of dynein functional-blocking antibody, clone 70.1, completely blocked the poleward movement of DNA beads (Figure 4A, arrow, n = 17, movie S2). To verify if the inhibition by anibody injection is due to specific disruption of dynein function, we performed separate experiment to inject dynamitin p50, a dynein inhibitory peptide [36] and observed the same inhibition of DNA bead poleward movement (Figure 4B, n = 23). The block was not due to possible activation of spindle assembly checkpoint which may arrest cell cycle progression because the maternal meiotic spindle in the same eggs underwent normal metaphase-anaphase transition and chromosome segregation (Figure 4A–4C, arrowheads). It is interesting to note that disruption of dynein function had no effect on the bipolar segregation of the meiotic chromosomes (Figure 4A and 4B, arrowheads) which contain kinetochores. The differential effects of inhibition of dynein on the behavior of kinetochore-free DNA beads and kinetochore-containing meiotic chromosomes after anaphase onset are summarized in Figure 4C.

To determine the possible site of action of dynein, DNA bead-injected eggs were stained with an antibody against the 74kD dynein intermediate chain. Immunostaining showed dynein localization on both DNA beads (Figure 4D and 4E, arrow), and kinetochores and spindle poles of the maternal spindle (Figure 4F and 4G, arrow and arrowheads), which is in agreement with previous reports [18], [19]. DNA bead-spindle poles, however, were not as clearly stained by dynein antibody as those of the maternal meiotic spindle (Figure 4D and 4F) and fewer foci of p-MARCKS-stained spindle poles were observed in DNA bead-spindles (Figure 4H, arrows) compared with that of the maternal MII spindle (Figure 4J, arrows). Accordingly, dynactin p150Glued, a functional partner of dynein consistently localized to DNA beads (Figure 4L, 4M) and meiotic chromosomes (Figure 4N, 4O). Taken together, the results suggest that dynein contributes to the chromatin poleward movement during anaphase.

### Disruption of microtubule dynamics blocks both DNA bead and meiotic chromosome movements toward the poles

**Figure 5:**
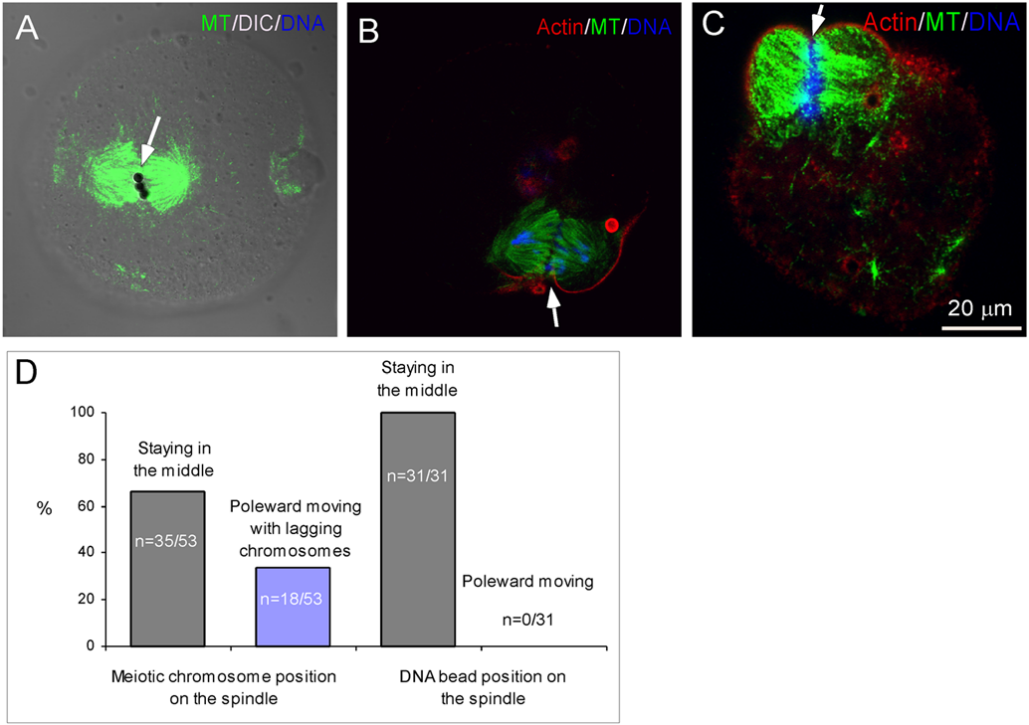
The effects of taxol on chromosome poleward movements during anaphase. (A) DNA beads (arrow) maintained at the equatorial position of the spindle after egg activation in the presence of 100 nM taxol. (B) The same egg shown in (A) showed anaphase onset and impaired poleward movement of maternal chromosomes. Note the cytokinetic furrow induced by the spindle midzone (arrow) and the lagging chromosomes. (C) Complete inhibition of chromosome poleward movement in a taxol-treated egg. Note that a cleavage furrow (arrow) is formed over the spindle midzone, suggesting that the egg did enter anaphase. (D) Quantification of chromosome poleward movements on both meiotic and DNA bead spindles in the presence of 100 nM taxol.

To test if microtubule depolymerization contributes to the observed DNA bead poleward movements, low concentrations of taxol (100 nM-10 µM) were used to block microtubule flux as previously reported [20], [37]. Poleward movements of both DNA beads and meiotic chromosomes were inhibited by taxol, resulting in DNA beads and most of the meiotic chromosomes staying at the equators of the spindles (Figure 5A–5C, arrows). A small percentage of eggs (18/53) showed partial inhibition of the maternal chromosome poleward movements with some lagging chromosomes at the equator regions (Figure 5B and 5D, arrows). The effects of taxol on DNA-bead and meiotic chromosome movements were unlikely due to cell cycle arrest as a result of possible checkpoint activation since typical telophase spindle morphology and anaphase spindle midzone-induced furrows were all observed in these eggs (Figure 5B and 5C, arrows), suggesting that cell cycle progression was unaffected by taxol treatment.

## Discussion

It was previously shown that a bipolar spindle can assemble around DNA beads in egg extracts, independently of kinetochores or centrosomes [28]. It has been unclear, however, if these DNA bead-spindles are functional in segregating chromosome and inducing cytokinesis in the live cells. Here, we report that despite lack of kinetochores and centrosomes, the DNA-bead-induced spindles are still able to generate poleward forces to move DNA beads to spindle poles during anaphase. In addition, the DNA bead-spindles are fully functional in inducing cytokinesis and result in ectopic polar body extrusion during meiosis II in mouse eggs. The observed poleward movements of DNA beads in the kinetochore-less spindle are not due to random movements of the beads because: 1) the poleward movements were not observed in the metaphase spindles and non-DNA-coated beads showed no poleward movements (data not shown); 2) the kinetochore-independent chromatin poleward movements were also observed in the sperm chromatin-assembled spindles which are known to contain no kinetochore fibers in the absence of DNA replication [35]. Thus, a chromatin-induced bipolar spindle formed in the meiotic oocytes has an intrinsic property to move chromosomes from the equator to poles at anaphase even in the absence of kinetochores.

The presence of kinetochores and their proper attachment to microtubules to establish a biorientation configuration may provide a more advanced structure to ensure accurate chromosome segregation while installing a checkpoint to monitor the process. In the absence of kinetochores, both DNA beads and sperm chromatin show random monopolar poleward movements, resembling the case of chromosome nondisjunction during the error-prone meiosis [38], [39]. The kinetochore-independent mechanism for chromosome poleward movements may contribute to the high rate of aneuploidy in mammalian meiosis [39]. However, it remains to be determined to what extent the results obtained from the artificially-assembled spindles can be applied to the kinetochore-containing meiotic spindles during meiosis. An interesting question that also needs to be addressed in the future is how the kinetochore-dependent and the kinetochore-independent poleward forces are coordinated during meiosis.

It was previously observed in plant cells and insect spermatocytes that microsurgically generated chromosome fragments, which contain no kinetochores, are able to move to spindle poles [20], [21]. However, in these experiments, due to the presence of both intact chromosomes and kinetochore-free chromosome fragments in the same spindle, it was unclear if the chromosome fragments could be “hijacked” by the kinetochore-microtubule generated poleward forces. Another difference is that the previously reported kinetochore-independent poleward movements were observed both at metaphase and anaphase, while the poleward movement of DNA beads described here is tightly coupled with anaphase onset, suggesting that the observed poleward force generation described here is under precise cell cycle control.

In mitotic cells, the onset of anaphase is triggered by cohesin degradation by separase [40], [41]. Artificial severing of the linkage between sister chromosomes during metaphase in mitotic cells could result in premature poleward movements of chromosomes [42], [43], [44], suggesting that the mitotic chromosomes are subject to persistent poleward forces during both metaphase and anaphase. However, in the meiotic oocytes used in this study, there is no DNA replication in the metaphase-arrested eggs and it is unlikely that chromatin cohesion is responsible for restraining poleward movements of DNA beads or sperm chromatin during metaphase. It is more likely that the observed poleward movements of DNA beads or sperm chromatin after anaphase onset are due to direct activation of motors or microtubule depolymerization-based forces as a result of the cell cycle transition.

There are two potential mechanisms by which the minus end-directed motor dynein could drive chromosome poleward movement: by moving the bulk of chromosomes as a cargo toward spindle poles, or by powering the microtubule poleward flux by affecting microtubule minus end dynamics at the spindle poles [28], [45]. The mictotubule poleward flux has been observed in meiotic spindles in Xenopus eggs [44], [46], [47]. We have observed dynein and p150Glued localized on the injected DNA beads and sperm chromatin, suggesting that dynein possibly directly transports the bulk of chromosomes toward the microtubule minus ends at spindle poles. However, a simple transport mechanism is unlikely, as microtubule depolymerization is also critical for poleward chromosome movement. It is interesting to note that although the movements of the DNA beads and the maternal chromosomes show different dependencies on dynein, the resultant rates of chromosome poleward movement are almost identical. One possible explanation for this phenomenon is that the same microtubule depolymerization event is rate-limiting for both types of movement.

## Materials and Methods

All animals were handled in strict accordance with good animal practice as defined by the National Institute of Health of the United States and guidelines of Institutional Animal Care and Use Committee (IACUC) and all the animal work was approved by the IACUC committee at Stowers Institute for Medical Research, protocol #2007-0013.

### Microinjection

Metaphase II (MII) arrested eggs were collected from mice after superovulation treatment as described [48]. To induce spindle formation by DNA beads or sperm chromatin, 3–5 beads coated with plasmid DNA [28], [29] or an inactivated sperm head were injected into eggs as described before [29], [48]. To induce formation of a single spindle around both DNA beads and sperm chromatin, DNA beads and sperm chromatin were co-injected into eggs. The DNA bead- and sperm chromatin-injected eggs were cultured in M16 (Chemicon) for 3–4 h to allow microtubules to assemble into a bipolar spindle.

To assess the role of dynein during chromosome poleward movements, an antibody against dynein intermediate chain, clone 70.1 (Sigma), was injected into eggs at a final intracellular concentration around 1 mg/ml before induction of anaphase (see below). Recombinant dynamitin p50 prepared as described [36] was injected at a final concentration around 0.4 mg/ml. To visualize DNA bead and meiotic chromosome poleward movements during anaphase in live eggs, rhodamine-labeled tubulin (Cytoskeleton) was injected into eggs at a final concentration of 0.1 mg/ml.

### Egg activation

To induce anaphase onset, eggs were activated parthenogenetically by culture in 10 mM SrCl_2_ in Ca^2+^, Mg^2+^ free CZB [32], [33] or by in vitro fertilization [34]. The anaphase chromosome poleward movements were evaluated 1.5 h after egg activation by fixation and immunofluorescence (see below).

### Live imaging of chromosome poleward movements and polar body extrusion during meiosis II

To observe chromosome poleward movements during anaphase, eggs containing both a DNA bead-spindle and a maternal meiotic spindle were injected with rhodamine-labeled tubulin as described above and activated with SrCl_2_. To visualize chromosomes, Hoechst33342 (Sigma) was added to the medium at concentration of 5–10 ng/ml. Confocal Z-series sections spanning both the DNA bead spindle and the meiotic spindle were collected on a Zeiss 510 NLO with a time interval of 7 min. During the course of imaging, 5% CO_2_ was supplied and temperature was maintained at 37°C.

### Immunofluorescence confocal microscopy analysis

Eggs were fixed in 3.7% paraformaldehyde and processed as described [29]. Microtubules were visualized by staining with a mouse beta tubulin antibody (1∶500) (Sigma). Kinetochore structure was detected by using a rabbit polyclonal CENP-A antibody (1∶200) (Abcam). A rabbit polyclonal survivin antibody (1∶250)(Abcam) was used to stain spindle midzone. Cytoplasmic dynein was stained by a mouse dynein antibody (1∶300) (74kd, Chemicon). Microtubule organization centers (MTOCs) were stained by a phospho-MARCKS (myristoylated alanine-rich C-kinase substrate) antibody (1∶400, Sigma). A mouse p150Glued antibody (BD Transduction Laboratory) was used to stain dynactin p150 (1∶300). Corresponding secondary antibodies conjugated with Alexa Fluor 488, 633 were used to reveal the primary antibody-staining (1∶400). F-actin was visualized by staining with Alexa Flour 546 conjugated phalloidin (Molecular Probes) at a concentration of 6.6 µM. DNA was counterstained by DAPI (Sigma) at a concentration of 1 µg/ml.

All images were acquired by using a 40× or 63× oil objective on a Leica confocal microscope or the Zeiss LSM510 confocal microscope. To construct 3D images and movies, a stack of 50 Z-section images spanning all the observed structures was collected and reconstructed using Leica confocal software and Zeiss LSM-CFS. The reconstructed Z-series images were exported as AVI movies to show 3D structures. For chromosome poleward motility analysis, the timelapse Z-stack images were analyzed using Imaris (Bitplane AG). The figures were assembled in Adobe Photoshop 7.0.

## Supporting Information

Figure S1 Time course of poleward movements of DNA beads and meiotic chromosomes during the anaphase of meiosis II. Microtubules are shown in red, DNA in green. DNA bead-spindle is at the 11 o'clock position and the meiotic chromosome/spindle is at the 4 o'clock position. The numbers in the red squares at the upper left corners indicate the timing.
(9.70 MB TIF)

Movie S1 Poleward movements of DNA beads and meiotic chromosomes during anaphase of meiosis II. Note the white circled eggs which show DNA bead (white arrows) poleward movement and polar body extrusion. Microtubules are shown in red, DNA in blue.
(15.73 MB AVI)

Movie S2 Disruption of dynein blocks DNA bead poleward movement (arrow) but not meiotic chromosome segregation. Microtubules are shown in green, actin in red and DNA in blue.
(29.10 MB AVI)
