# Supplementary figures and images for: Kinetochore-Independent Chromosome Poleward Movement during Anaphase of Meiosis II in Mouse Eggs
Source: PLoS One. 2009 Apr 13;4(4):e5249. doi: 10.1371/journal.pone.0005249 (PMC2664963; doi:10.1371/journal.pone.0005249)

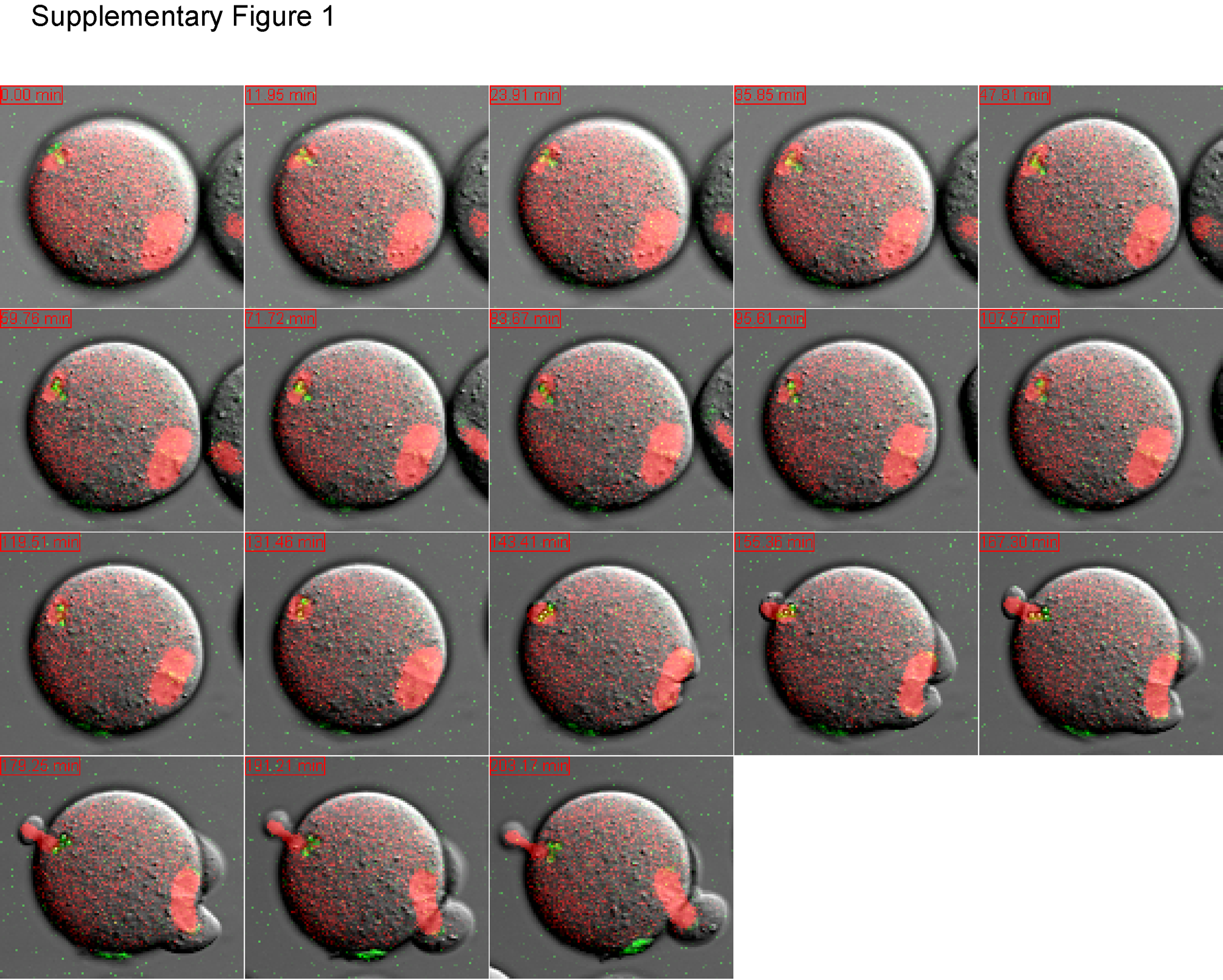

Supplement: Figure S1 — Time course of poleward movements of DNA beads and meiotic chromosomes during the anaphase of meiosis II. Microtubules are shown in red, DNA in green. DNA bead-spindle is at the 11 o'clock position and the meiotic chromosome/spindle is at the 4 o'clock position. The numbers in the red squares at the upper left corners indicate the timing. (9.70 MB TIF) [file pone.0005249.s001.tif]
